# A robust, autonomous, volumetric quality assurance method for 3D printed porous scaffolds

**DOI:** 10.1186/s41205-022-00135-x

**Published:** 2022-04-06

**Authors:** Nicholas Zhang, Srujan Singh, Stephen Liu, Wojciech Zbijewski, Warren L. Grayson

**Affiliations:** 1 Department of Biomedical Engineering, Translational Tissue Engineering Center, Johns Hopkins University, 400 North Broadway, Smith Building 5023, Baltimore, MD 21231 USA; 2 Translational Tissue Engineering Center, Johns Hopkins University, Baltimore, MD USA; 3 Department of Chemical and Biomolecular Engineering, Johns Hopkins University, Baltimore, MD USA; 4 Department of Materials Science and Engineering, Johns Hopkins University, Baltimore, MD USA; 5 Institute for Nanobiotechnology, Johns Hopkins University, Baltimore, MD USA

**Keywords:** 3D printing, Scaffolds, Bone tissue engineering, Quality assurance, Iterative closest points

## Abstract

Bone tissue engineering strategies aimed at treating critical-sized craniofacial defects often utilize novel biomaterials and scaffolding. Rapid manufacturing of defect-matching geometries using 3D-printing strategies is a promising strategy to treat craniofacial bone loss to improve aesthetic and regenerative outcomes. To validate manufacturing quality, a robust, three-dimensional quality assurance pipeline is needed to provide an objective, quantitative metric of print quality if porous scaffolds are to be translated from laboratory to clinical settings. Previously published methods of assessing scaffold print quality utilized one- and two-dimensional measurements (e.g., strut widths, pore widths, and pore area) or, in some cases, the print quality of a single phantom is assumed to be representative of the quality of all subsequent prints. More robust volume correlation between anatomic shapes has been accomplished; however, it requires manual user correction in challenging cases such as porous objects like bone scaffolds. Here, we designed porous, anatomically-shaped scaffolds with homogenous or heterogenous porous structures. We 3D-printed the designs with acrylonitrile butadiene styrene (ABS) and used cone-beam computed tomography (CBCT) to obtain 3D image reconstructions. We applied the iterative closest point algorithm to superimpose the computational scaffold designs with the CBCT images to obtain a 3D volumetric overlap. In order to avoid false convergences while using an autonomous workflow for volumetric correlation, we developed an independent iterative closest point (I-ICP_10_) algorithm using MATLAB®, which applied ten initial conditions for the spatial orientation of the CBCT images relative to the original design. Following successful correlation, scaffold quality can be quantified and visualized on a sub-voxel scale for any part of the volume.

## Introduction

Three-dimensional (3D) printing has emerged as an effective tool for use in bone tissue engineering approaches due to its versatile fabrication capabilities. Porous, rigid scaffolds used to treat critical-sized craniofacial bone defects involve complex macroscopic geometries to recapitulate the structure of facial bone. Scaffolds may also have heterogeneous microscopic architectures to vary scaffold stiffness based on predicted load distribution while simultaneously facilitating cellular and vascular infiltration post implantation [1]. The application of 3D printing technologies enables the fabrication of customized scaffold designs for unique patient defects. However, the customized nature of these defects presents challenges in the context of Good Manufacturing Practice where the print quality of each individual scaffold needs to be assessed.

The quality assurance (QA) assessments presented in the tissue engineering literature have thus far been limited to regular geometries such as cylinders and pyramids [2, 3]. Furthermore, they rely on scalar values or one and two dimensional metrics [3, 4] to describe increasingly complex 3D scaffolds. More comprehensive 3D QA metrics have been accomplished but require manual input in difficult cases such as porous objects [5]. In the laboratory setting, fabrication quality is typically characterized by qualitative methods that include scanning electron microscopy (SEM), and stereoscopic or brightfield imaging, and quantitative techniques such as computed tomography (CT) scans. CT scanning has been noted to be the best non-destructive characterization method based on its ability to capture large 3D volumetric structures at relatively high resolution, but there is a need for better algorithms to process the data and compare 3D-print outcomes to the theoretical design [6]. Beyond tissue engineering applications, QA assessments for 3D printing typically involve fabricating phantoms with precise and regular geometries, which can be easily validated by measuring with calipers [7]. The premise of this approach is that the deviation measured in the phantom is representative of that occurring in all subsequent prints. This method provides macroscale QA for a particular 3D printer at a single point in time and is unsuitable for assessing porous microstructures.

The Iterative Closest Points (ICP) is a well-established method of registering 3D volumes [8, 9]. It is a widely used method in fields like remote sensing, computer vision, and robotics. However, ICP is sensitive to initial position of the volumes, which can result in false registration. To overcome this limitation, several groups have proposed additions [10] to the traditional ICP algorithm by accounting for unique geometric features [9], spherical harmonics, Gaussian based image registration, and invariant features of the two volumes [11]. However, these methods either involve some degree of manual input or may still result in false registration.

**Fig. 1:**
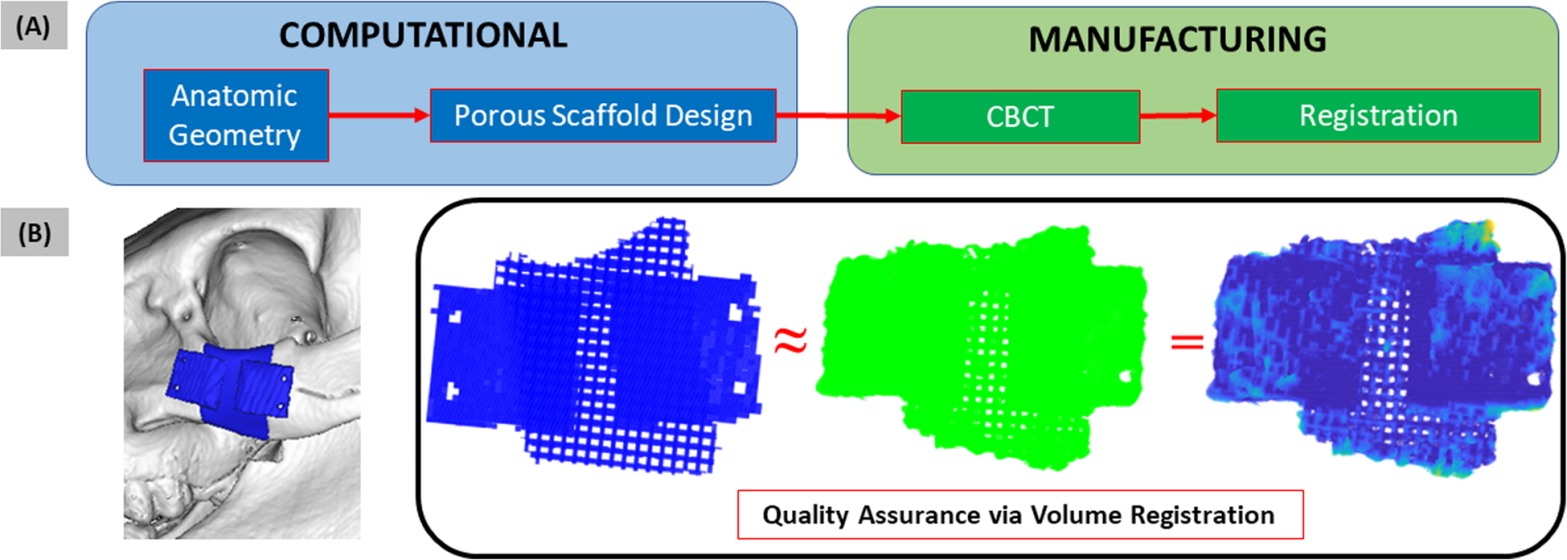
Patient-specific tissue engineering scaffolds require robust quality assurance metrics. (**A**) General workflow for design and manufacture of custom, porous bone scaffolds. Solid scaffold geometry is generated from anatomic geometry of defect site. (**B**) Custom design and manufacture of 2 cm porcine zygomatic porous scaffold to treat the shown defect. Solid scaffold is assigned a custom porous pattern (blue). The design is 3D printed and scanned. CBCT scan (green) of manufactured scaffold provides its digital representation. Subsequent QA involves 3D correlation between manufactured scaffold and design intent to produce quality readout

Thus, there is a need for an autonomous, 3D QA method capable of processing complex scaffold geometries with heterogeneous porosities. This will be paramount for a robust QA framework to match quality thresholds of standard manufacturing, which is necessary for commercial translation from lab to clinic [12]. Ideally, this method should involve volume correlation (‘registration’) between manufactured scaffold and design intent to quantify spatial similarity on a sub voxel scale (Fig. 1). In this study, we aim to demonstrate an autonomous, 3D, quantitative QA pipeline for anatomically matched, heterogeneous tissue engineering scaffolds with varying complexity. First, we achieve successful volume registration autonomously for various 3D printed scaffolds. Then, we use the successful registration to measure and visualize areas of fabrication error.

## Materials and methods

### Design of anatomic scaffolds

Two anatomic shapes – human zygoma and orbital arch – were designed in Mimics (Materialise Mimics 14.0®, Leuven, Belgium). These designs were exported in image (.bmp) slices and imported into MATLAB® using a custom script to recreate the 3D volume in matrix format (.mat). The cylinder volume with 25 mm diameter and 50 mm height was generated directly in MATLAB. To make these 3 volumes porous, open source scafSLICR [13] (MATLAB Central File Exchange) was used to customize porosity and slice the design for 3D printing. First, each scaffold was created with homogeneous porosity with pore width of 0.8 mm. The sliced output (.gcode) was saved for 3D printing. This process was repeated to create the same designs but with a heterogeneous porosity using a linear gradient of clinically-relevant pore widths – 0.2, 0.4, 0.6, 0.8, and 1 mm. The new sliced outputs (.gcode) were saved for 3D printing.

### 3D printing and scanning of scaffolds with various geometry and porosity

All 6 scaffolds were 3D printed with a Lulzbot Taz 5 (Aleph Objects) using the gcode files. Acrylonitrile butadiene styrene (ABS; IC3D Industries, Columbus, Ohio) filament was fed into the printer. After printing, all 6 scaffolds were scanned using cone beam computed tomography (CBCT; Carestream OnSight3D). Typically, it provides high quality renders at sub-millimeter resolution and acquisition times of about 1 minute [14]. Scans were acquired at 0.139 mm detector pixel size, 420 projection views over 216^o^, 10 mA tube current, and 70 kVp tube potential. Subsequently, scans were reconstructed at 0.26 mm voxels. The CBCT dicom files were imported into Mimics. The region of interest was segmented out at approximately − 1000 to − 200 HU and cropped in 3D to the sample volume.

### Registration of scaffold design and 3D prints using I-ICP_10_

A custom script, I-ICP_10_, was written to import a scaffold design (“fixed volume”) and the CBCT scan of its 3D print (“moving volume”) in the form of a 3D matrix and convert them to a point cloud. The moving volume was rotated independently at angles of 90^o^, 180^o^, and 270^o^ about each x, y, and z axis from the center of mass (origin). Both volumes were downsampled with a box grid filter of 1 × 1 × 1 mm: for a moving box of width 1 mm, all the voxels were reduced to a single voxel at the average coordinates of those voxels. A box size of 1 mm is suitable for scaffolds on the order of a few cm because it reduces the number of voxels while still retaining gross geometry and porous pattern. In this manner, the registration process speed would drastically increase while also maintaining geometry. For each rotation, the traditional ICP registration algorithm was implemented using MATLAB’s Computer Vision Toolbox. The algorithm was terminated when the average of the differences in the previous 3 iterations yielded a translation less than 0.01 mm and a rotation less than 0.05^o^.

Upon termination for each rotation, the root mean squared error (RMSE) was calculated for the final conformation between the fixed and moving volumes by taking the squared Euclidean distance between corresponding voxels, averaging the distance for all pairs of voxels, and applying the square root of that average. This final scalar metric was taken to be indicative of the success level of the registration. The initial rotation that yielded the smallest final RMSE was considered the most successful registration and thus the optimal initial condition.

Although the ICP trials run on downsampled volumes provided approximately correct registration, the traditional ICP algorithm was executed a final time using the optimal initial rotation on the original, not downsampled, volumes. By using the original volumes, ICP could perform the last fine registration transformations necessary to find the most optimal registration possible. All scripts were executed on a 16 GB RAM Windows 10 laptop with 10th generation Intel Core i7 and Nvidia GeForce GTX 1650 Ti graphics card.

### Quality assurance outputs from successful registration

Once successful registration was autonomously achieved by the algorithm, a heatmap was generated for the CBCT volume in which the value of each voxel represented its deviation – ‘voxel error’ – from the design intent. The histogram of all voxels with their associated error was also plotted. Furthermore, each voxel’s associated error was plotted against its distance to the scaffold center of mass. Similarly, the scaffold was skeletonized into a single voxel thread throughout the entire volume. Then, the voxel error was plotted against its distance to the closest strut skeleton.

## Results

### Traditional ICP provided successful 3D registration of MIMICS-based porous scaffold designs with CBCT scans

**Fig. 2:**
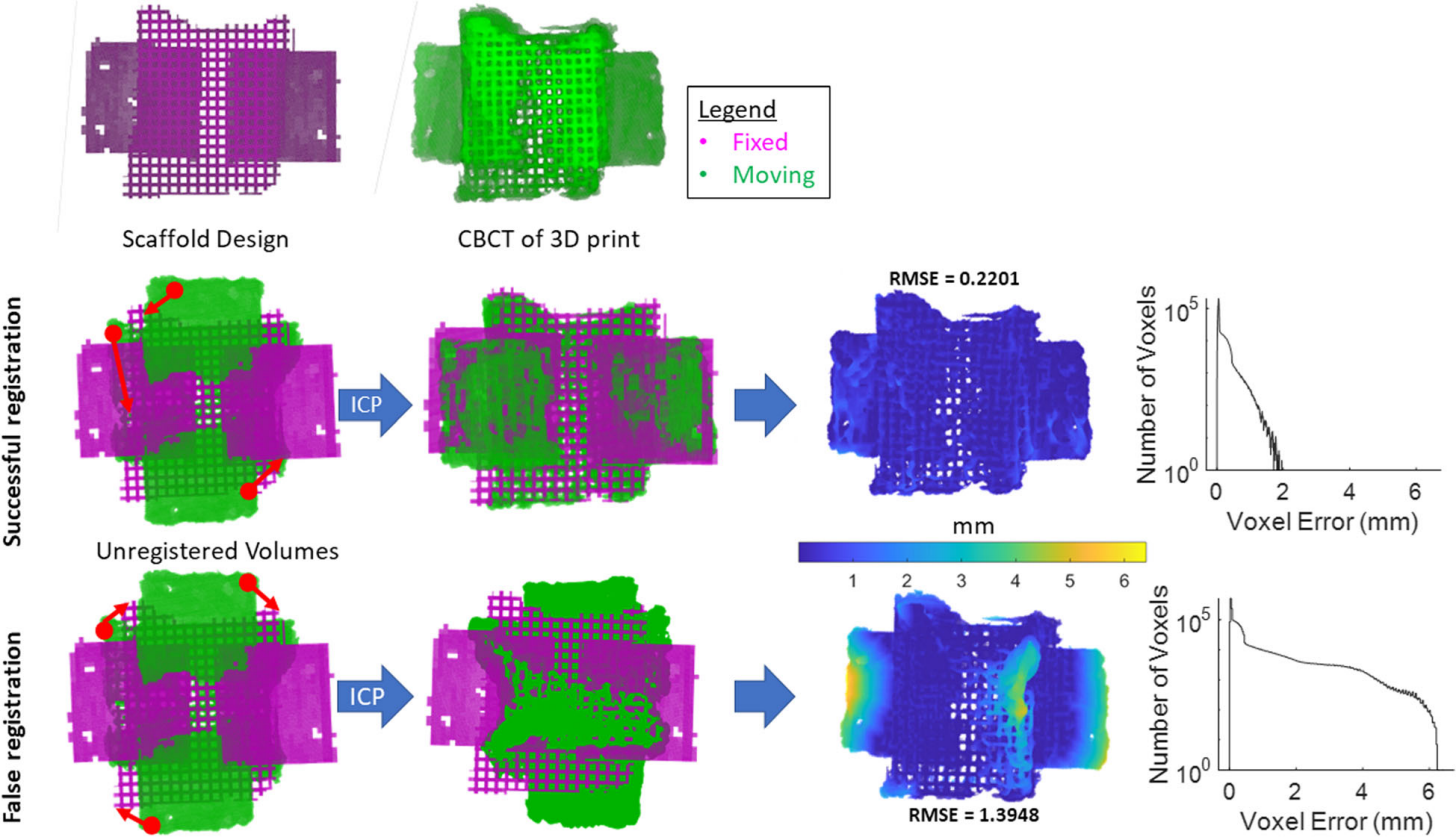
3D volume registration using ICP led to false convergences. (Top Row) Example porous scaffold (design intent; “fixed”) and CBCT scan of 3D printed scaffold (“moving”). Registration aims to calculate the optimal alignment between moving and fixed volumes. (Middle Row) ICP calculated optimal rigid transformations in 3D space between moving and fixed volumes. Once registered, the moving volume’s spatial error on a sub-voxel scale was measured and visualized in a heatmap. A histogram of all voxel error was plotted with most voxel error falling below 2 mm difference. (Bottom Row) ICP is sensitive to initial configurations and susceptible to false convergences in which large overlapping areas can trick the algorithm into classifying the registration as “successful”. In these cases, significantly higher voxel error was observed at the scaffold edges. As seen in the histogram, the voxel error had a much wider distribution, and the RMSE value for the whole scaffold is higher than in the successful scenario

Traditional ICP was robust and efficient for calculating the optimal registration between the CBCT of the 3D printed, porous scaffold (“moving”) and its design intent (“fixed”) (Fig. 2**Top-Middle**). Once successfully registered, the spatial error for every CBCT voxel was visually represented in a heatmap at sub-voxel scale, or in a histogram of all voxel error values. The RMSE value of the CBCT was calculated. However, depending on initial conformation of the volumes, ICP reached false registration states in which large portions overlap but gross geometries were still misaligned (Fig. 2**Bottom**). Misaligned portions exhibited much higher voxel error values on the heatmap, and the histogram revealed a much wider distribution of voxel error. Consequently, the RMSE value for the false registration was higher than that of the successful registration validating the algorithm used to autonomously identify the optimal registration.

### Using multiple initial rotations autonomously identifies optimal ICP registration (I-ICP_10_)

**Fig. 3:**
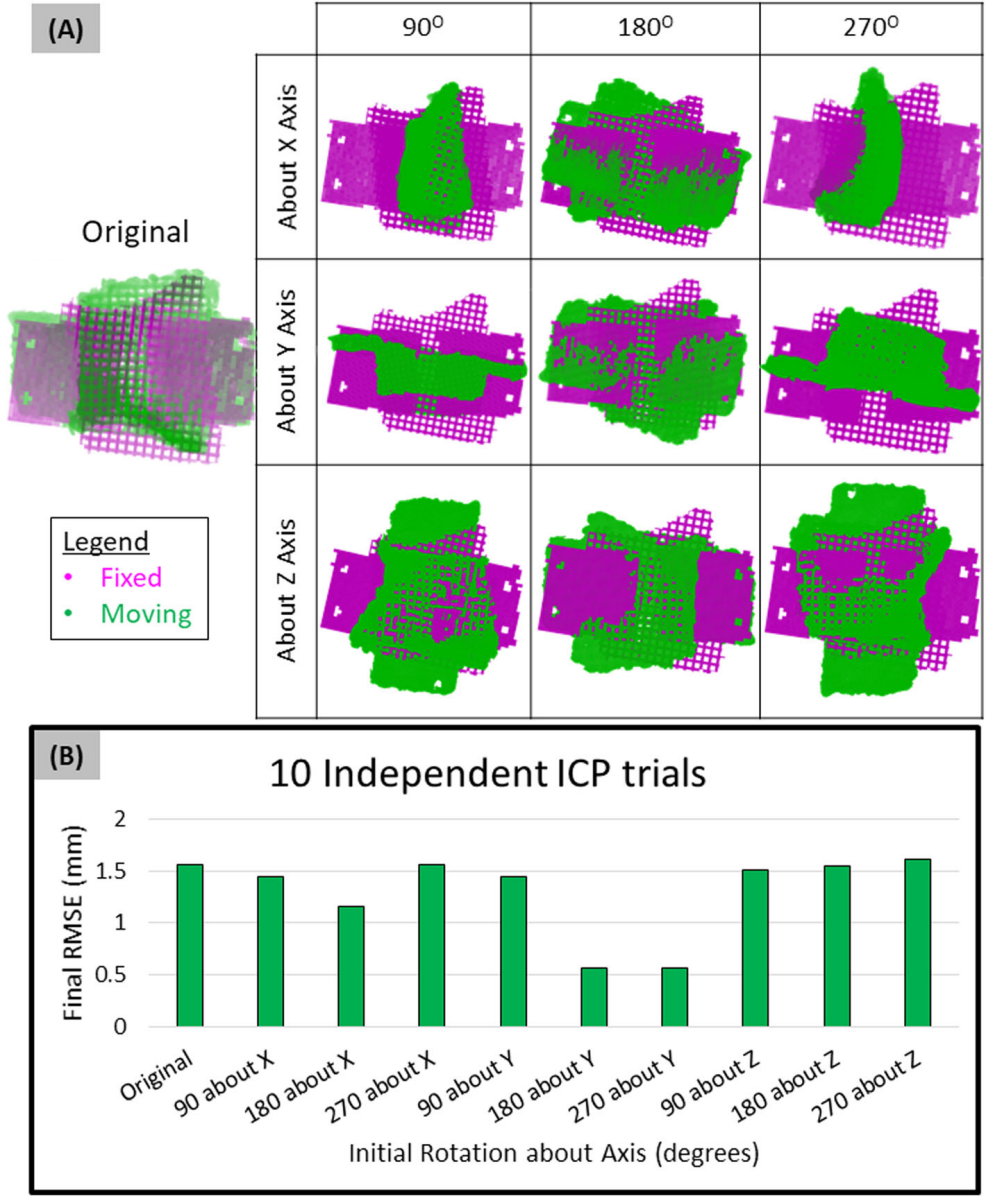
I-ICP_10_ workflow for QA of tissue scaffolds. (**A**) Execution independent ICP trials, each with varying initial rotations were applied to original moving volume. (**B**) ICP trials with the smallest final RMSE value were autonomously chosen as the most successful registrations. In this example, 180^O^ about Y and 270^O^ about Y were the favorable initial rotations that yielded successful registration

The initial orientation of the moving volume is chosen arbitrarily. Ten independent ICP trials (I-ICP_10_) were conducted by rotating the moving volume at 90-degree intervals about the Cartesian axes. Irrespective of the scaffold geometry, this was sufficient for the script to identify the favorable initial rotations that yielded successful registration based on RMSE of each trial result (Fig. 3). In this scenario, the 180^o^ about Y and 270^o^ about Y rotations yielded the smallest final RMSE (0.56 mm) from ICP. Visual inspection confirmed the same moving volume orientation for both of these trials. In fact, several different rotations yielded the same final RMSE from ICP – Original and 270^o^ about X (1.56 mm), 90^o^ about X and 90^o^ about Y (1.44 mm). Visual inspection further confirmed that these trials reached the same final conformation of the moving volume, suggesting that trials with the same RMSE were reaching the same final registration states.

### I-ICP_10_ was successfully applied to regular and irregular geometries with heterogenous porous microarchitectures

**Fig. 4:**
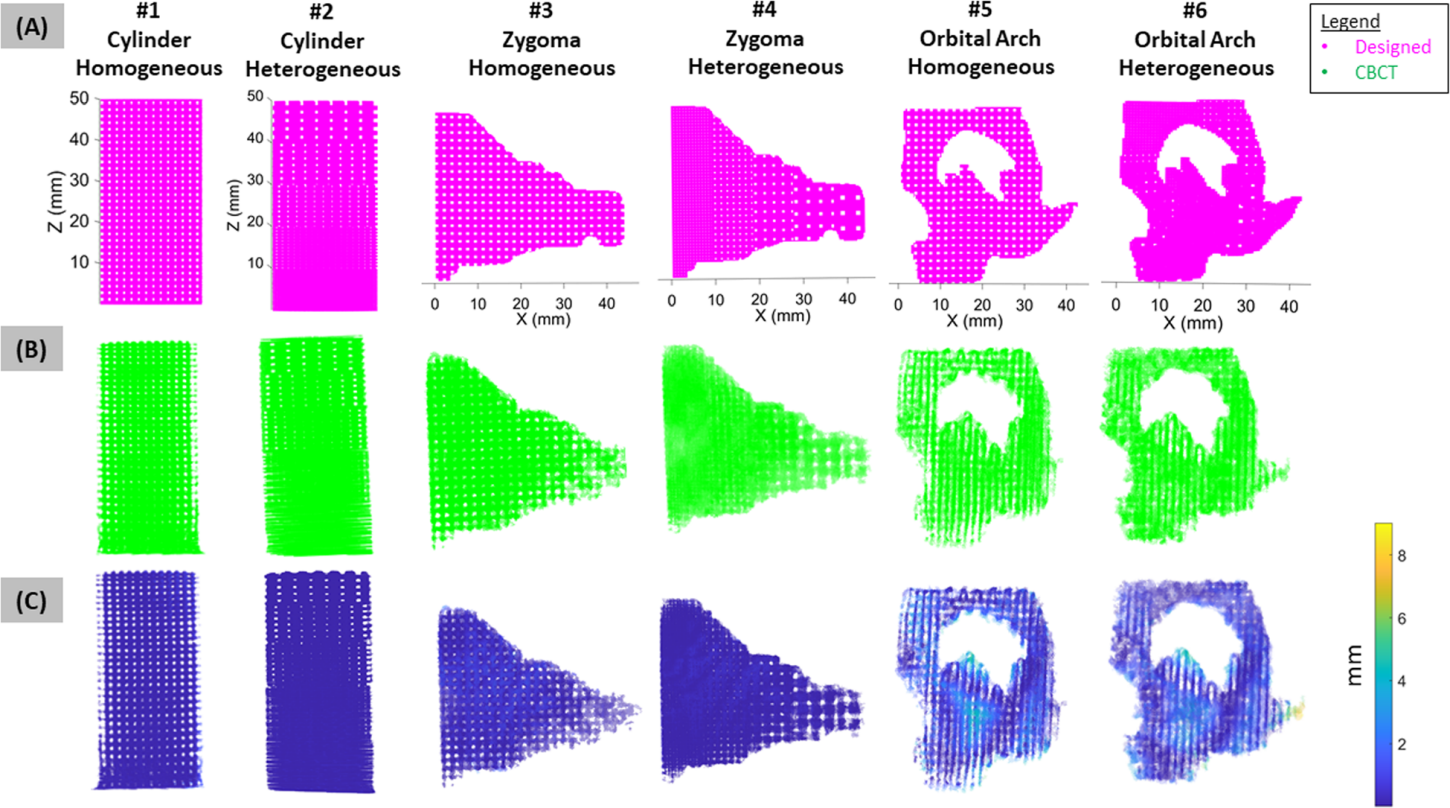
Validation of QA workflow on 3D printed scaffolds with varying complexity and porosity. (**A**) Six scaffolds were designed with various porosities (homogeneous or heterogeneous) and external geometries (cylinder, human zygoma, or human orbital arch). (**B**) Successful autonomous registration was achieved for all 6 scaffolds. (**C**) Using successful registration, a heatmap of voxel-to-voxel error for each scaffold was generated where each voxel’s deviation from the design intent was observed. The orbital arch exhibited noticeably worse voxel error than simpler geometries such as the cylinder

**Fig. 5:**
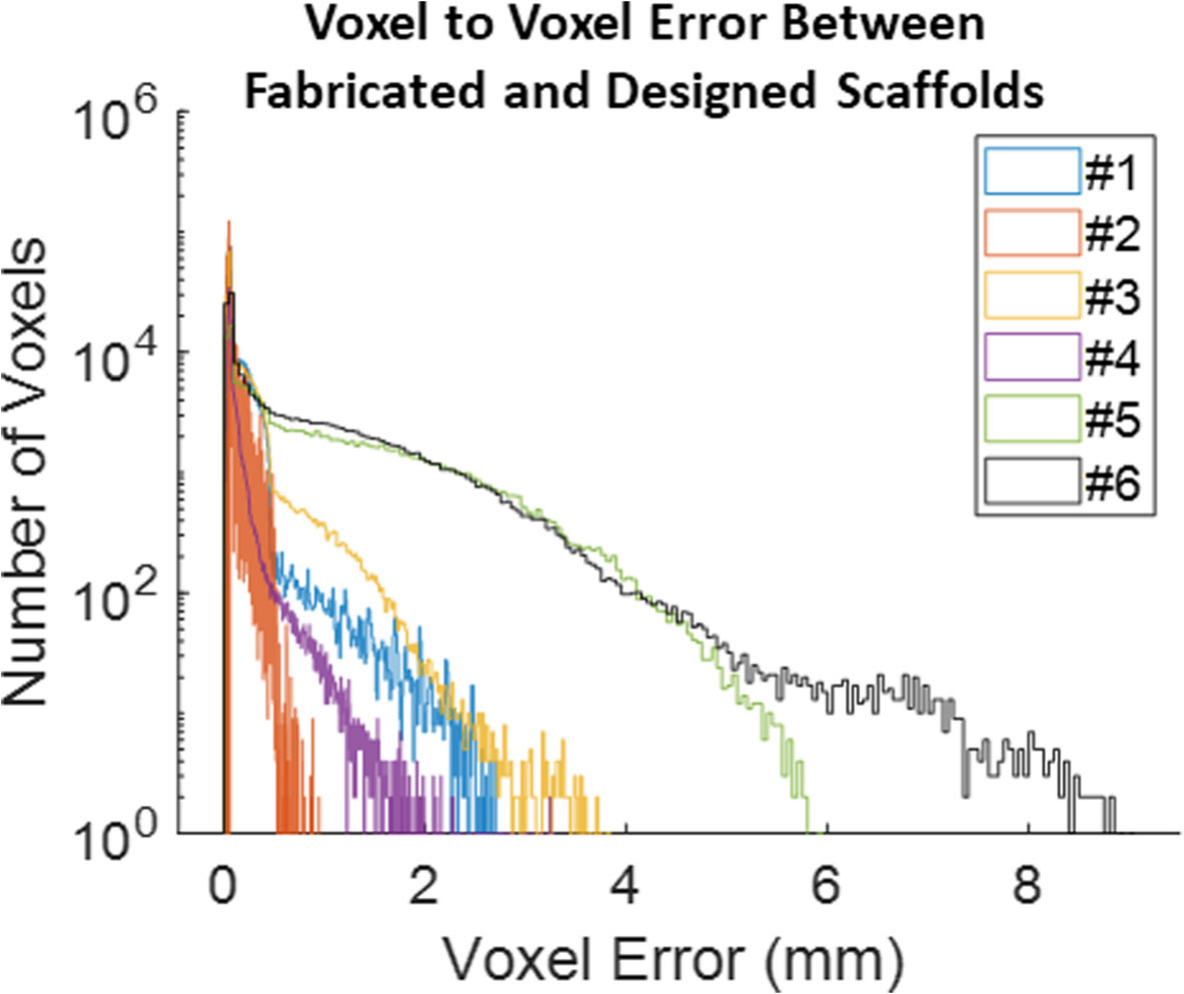
Quantification of 3D print quality for all voxels in 6 different scaffolds. Scaffolds with more complex exterior geometry (e.g. orbital arch) exhibited worse print quality, or larger voxel error, than simpler geometries (e.g. cylinder). Typically, scaffolds with heterogeneous porosities exhibited better print quality than their homogeneous counterparts except for the orbital arch. Overall, 3D print quality was captured for the entire scaffolds on a sub-voxel scale while yielding the general pattern of manufacturing error

Successful registration was autonomously attained within 2 min using I-ICP_10_ for scaffolds of varying porosity and complexity (Fig. 4A-C). Successful registration was visualized in the heatmap of voxel spatial error in relation to the design intent (Fig. 4C), which indicated specific areas of fabrication error, such as the thin bony structures of the orbital arch. Scaffolds with more complex geometry like the orbital arch exhibited worse print quality than scaffolds with simpler geometries like the cylinder (Fig. 5), which is likely attributed to the higher number of nozzle direction changes for complex compared to simpler geometry. For the cylindrical and zygoma geometries, the heterogeneous porous patterns yielded better print quality values than their respective homogeneous porous patterns (Fig. 5). The orbital arch scaffold was the exception to this pattern which is likely due to the thin bone structure on the homogeneous orbital scaffold that did not appear in the CBCT scan (Fig. 4B-C).

### I-ICP_10_ revealed spatial distribution of errors in scaffold print quality

**Fig. 6:**
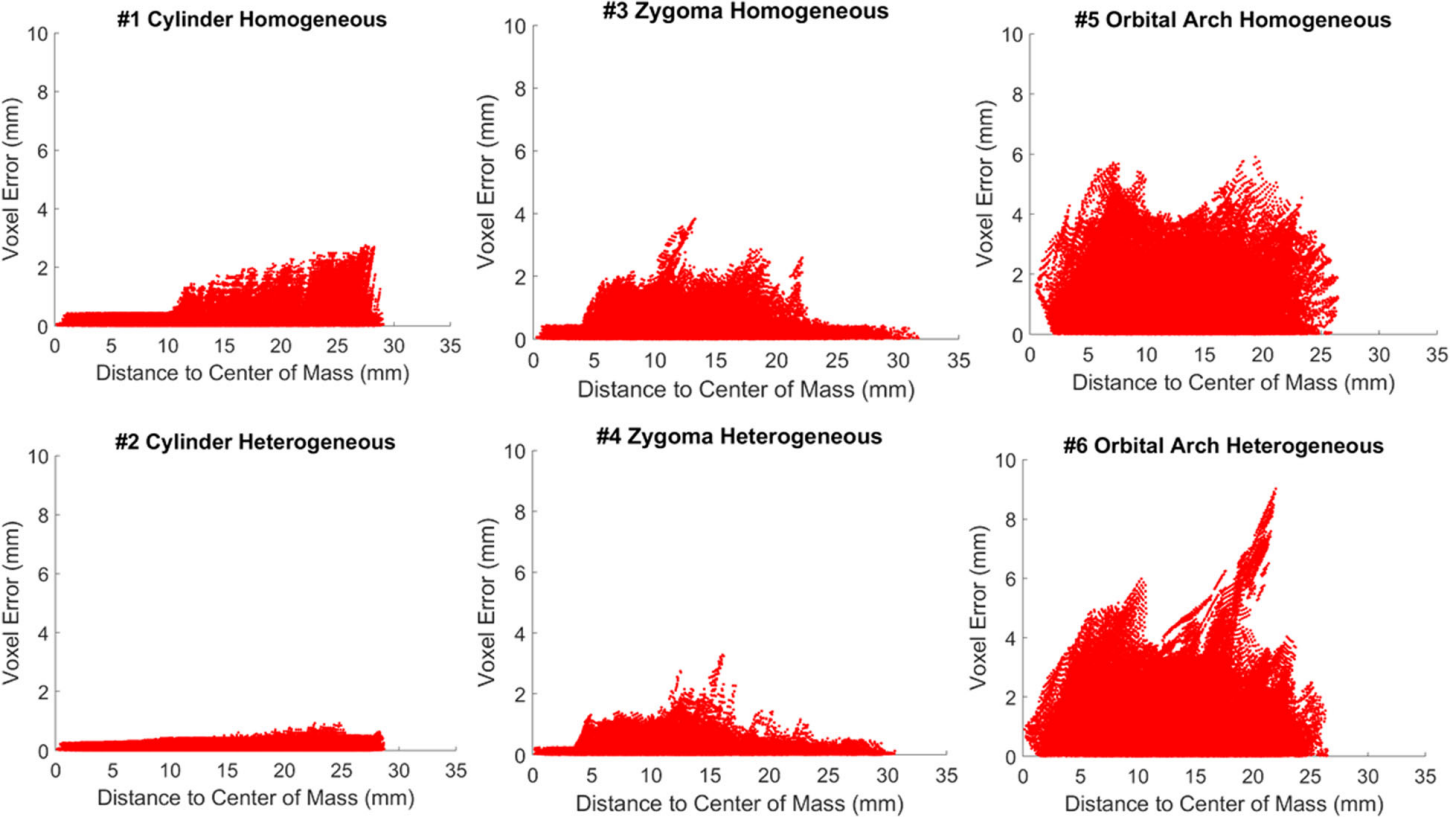
Voxel to voxel error compared to center of mass for all 6 scaffolds. Scaffolds exhibited noticeably worse print quality on their exterior surface where the 3D printer made abrupt changes in direction when depositing material. Note that for irregular geometries like the zygoma and orbital arch, the exterior boundary was not always the farthest distance from the center of mass. Additionally, scaffolds with more complex external geometries exhibited larger voxel error

**Fig. 7:**
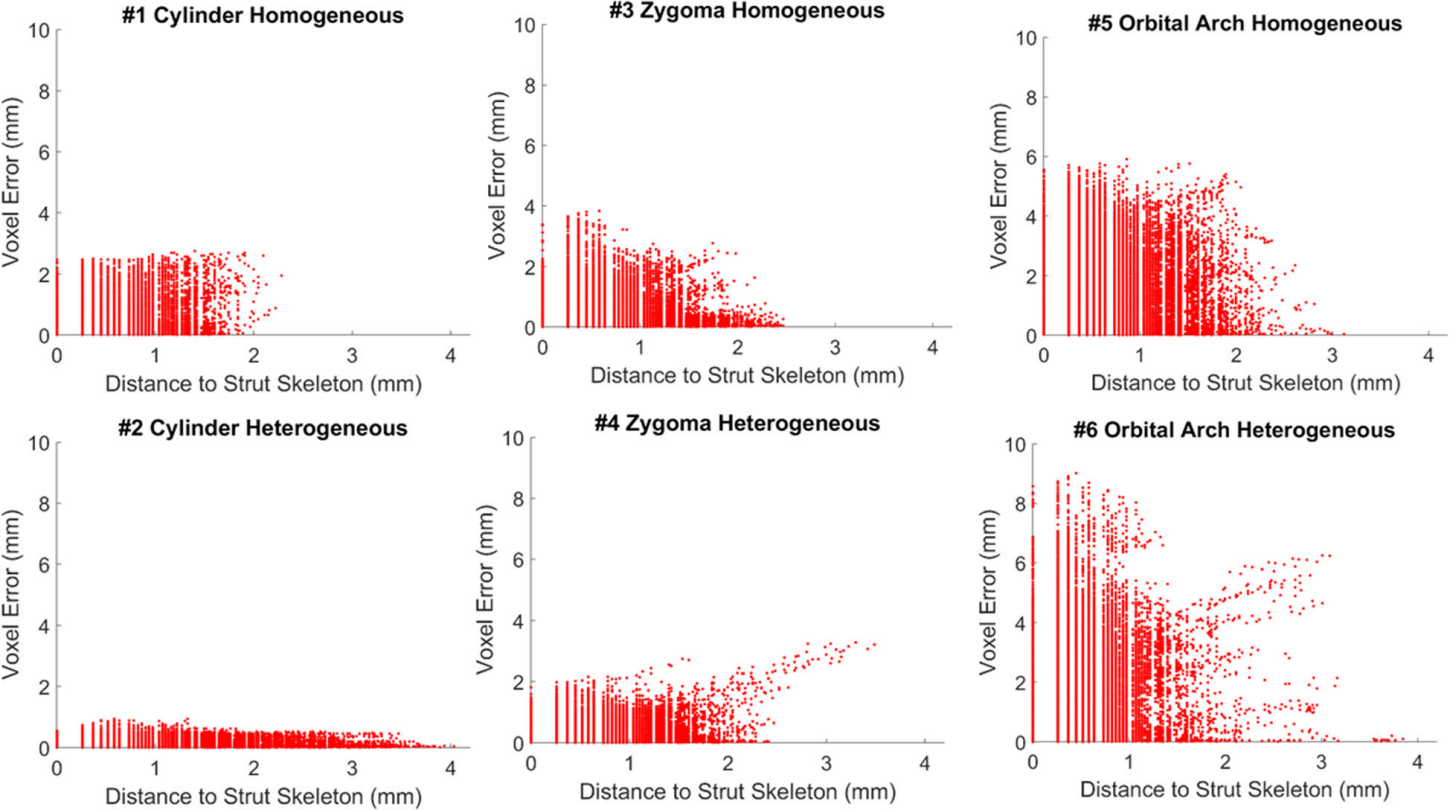
Voxel to voxel error compared to strut center (skeleton) for 6 scaffolds. Gaps between vertical lines approximated the fiber extrusion width of the 3D printer nozzle. Scaffolds with homogeneous porosities had more uniform gaps than their heterogeneous counterparts due to their more uniform strut widths. Thicker struts, or more voxels located farther from the skeleton, were observed for heterogeneous scaffolds over their homogeneous counterparts due to changing pore sizes. Voxels farthest from the strut skeleton lie on the exterior boundary and thus exhibited more random pattern than the vertical lines seen closer to the skeleton. Exterior voxels was likely support material from 3D printing that was not completely removed

Further spatial error distribution was observed. Scaffolds exhibited worse print quality on their exterior than their interior (Fig. 6; note that scaffold exterior may not necessarily be the farthest from the center of mass due to their irregular geometries). Again, in agreement with Fig. 5, higher voxel error values were positively associated with more complex geometries (Fig. 6). Every voxel’s associated error was also plotted against the strut skeleton of each scaffold (Fig. 7). The distance between the vertical lines was likely associated with the fiber extrusion widths of the printer nozzle. The presence of thicker struts, or more voxels farther from the strut skeleton, was observed for the heterogeneous scaffolds than their respective homogeneous counterparts, which was expected due to the changing pore sizes. The farthest voxels from the skeleton that did not seem to fall into a vertical line like the rest were attributed to the exterior of the scaffold, especially small support material that was not removed from the scaffold.

## Discussion

Over the past decade, 3D printing has become a powerful tool for fabricating custom bone scaffolds [15–19]. The versatile material and geometric capabilities of fused deposition modelling (FDM), for example, enable the production of porous, mechanically strong scaffolds of various material compositions [17]. Furthermore, its ability to rapidly manufacture precise macro and micro architecture makes it very suitable for treating a wide range of patient-specific defects.

Although the relationship between geometrical accuracy and bone regeneration is not well understood, the accuracy can affect scaffold mechanics which can then affect regeneration potential [17]. In a standard manufacturing setting, where reproducible parts are fabricated at mass scale, quality control is critical for verifying product performance and function. Similarly, for one off units such as bone scaffolds to treat bone loss in defect-specific manner, there is arguably a greater need for non-destructive characterization methods because each unit has unique architecture [6, 20]. Such methods should ideally be quick to implement and capture the complex 3D nature of these porous scaffolds at high resolution. Our method, I-ICP_10_, has been shown to meet these criteria for a wide range of scaffolds.

To date, bone tissue engineering studies have measured fabrication quality of 3D scaffolds in only one and two dimensions. For example, scaffold surface quality has been inspected with scanning electron microscopy (SEM) [21], which is only qualitative. More quantitative methods have involved 3D scans such as CT, but are processed minimally – measurements are typically limited to strut widths, pore widths, or global porosity percentages to describe a 3D scaffold [4, 21, 22]. In fact, 3D printing parameters and workflows have been optimized based on strut width [21], which is still a one dimensional metric. Such metrics are suitable only if the scaffolds are homogeneous; however, scaffold designs are becoming increasingly complex and heterogeneous [1], and thus require more robust 3D QA. General 3D printing workflows typically rely on precisely designed phantoms to gauge print quality at a certain point in time [7]. However, they are incapable of providing QA for any patient-specific scaffold. Such limitations become readily apparent as scaffolds translate from laboratory to clinic where they will need to be produced in larger quantities and characterized for QA quickly and efficiently.

General ICP was chosen as the base algorithm for our workflow due to its speed, easy implementation, and ability to handle large amounts of data [9]. Other registration algorithms exist and attempt to extract unique information from the volumes to improve registration accuracy in minimal time [10] but require larger computational expense, are less autonomous, and likely experience difficulty handling complex porous volumes, which are popular in bone tissue engineering. Future studies may directly compare the performance of I-ICP_10_ to existing registration algorithms with porous scaffolds.

The I-ICP_10_ workflow described here is heavily dependent on the scanning of the 3D printed scaffold via CBCT. CBCT is an expensive and unique equipment, and thus alternatives should be explored. The imaging modality should ideally be high resolution like CT, but quick to implement for acquisition and reconstruction of the 3D scaffold. Because bone scaffolds contain pore sizes in the range of 100–500 μm^15^, the imaging resolution need to be in the 50–80 μm range. I-ICP_10_ may still successfully register scaffold volumes at lower resolution but yield worse-than-actual readouts of print quality. High resolution micro CT (μCT) has been noted to be one of the best non-destructive characterization methods that needs better processing algorithms [6], which I-ICP_10_ could fulfill. The imaging modality will also depend on the material of the scaffold being scanned. In this study, scaffolds were printed using ABS for ease and cost compared to other customized materials used in our previous studies. CBCT was a suitable scanning method for ABS. Higher contrast and/or resolution may be needed depending on the material composition. Future studies will validate the workflow for more traditional biodegradable materials used in bone regeneration. Another improvement could be made by using alternative imaging modalities such as Keyence© scanners and comparing their performance to CBCT using I-ICP_10_. Regardless of image modality, I-ICP_10_ only requires a voxel mesh (“point cloud”) input of the 3D printed scaffold to successfully output print quality.

Ironically, in this study, the cylinder was the most difficult geometry to register successfully due to its near infinite planes of symmetry and thus false registration state(s). In a clinical setting, such a regularly shaped anatomic defect is unlikely to occur. Thus, we do not anticipate this challenge to be relevant in tissue engineering applications. Future work will be focused on using the objective readouts from this study to establish quality thresholds for manufacturing dependent on clinical use case i.e., type of tissue being regenerated. Additionally, longer term work can use the quality readouts shown in this study to create quality control feedbacks where necessary adjustments are made to manufacturing inputs, such as g-code instructions for 3D printers, to correct areas of fabrication error.

## Conclusion

Current fabrication quality metrics for bone tissue engineering scaffolds are too rudimentary for the mass manufacture of complex, heterogeneous, patient-specific geometries. Prior to implantation, each scaffold should be subjected to robust QA that can successfully handle its 3D and porous nature, identify areas of significant fabrication error, and objectively quantify it in a timely manner. In this study, we have successfully demonstrated a robust, 3D volume correlation method (I-ICP_10_) in MATLAB capable of quantitatively capturing the manufacturing quality for tissue engineering scaffolds with various external geometry and porous complexity. I-ICP_10_ involves minimal time – less than 2 min to register each scaffold – and computational expense – single threaded program on a 16 GB memory laptop. Moreover, our method does not rely on proprietary software and is completely autonomous.

## Data Availability

MATLAB data and scripts are available upon request from zhang.nicky4@gmail.com.
